# Attachment: the mediating role of hope, religiosity, and life satisfaction in older adults 

**DOI:** 10.1186/s12955-021-01695-y

**Published:** 2021-02-15

**Authors:** Saeed Pahlevan Sharif, Mohammadreza Amiri, Kelly-Ann Allen, Hamid Sharif Nia, Fatemeh Khoshnavay Fomani, Yasaman Hatef Matbue, Amir Hossein Goudarzian, Sedigheh Arefi, Ameneh Yaghoobzadeh, Hassam Waheed

**Affiliations:** 1 Taylor’s Business School, Taylor’s University, No.1, Jalan Taylor’s, 47500 Subang Jaya, Selangor Malaysia; 2 School of Pharmacy, Faculty of Health and Medical Sciences, Taylor’s University, 47500 Subang Jaya, Selangor Malaysia; 3 The Faculty of Education, Monash University, Clayton, Australia; 4 School of Nursing and Midwifery Amol, Mazandaran University of Medical Sciences, Sari, Iran; 5 School of Nursing and Midwifery, Tehran University of Medical Sciences, Tehran, Iran; 6 Qazvin University of Medical Sciences, Qazvin, Iran; 7 Student Research Committee, Mazandaran University of Medical Sciences, Sari, Iran; 8 Faculty of Medical Sciences, Tarbiat Modares University, Tehran, Iran; 9 Tehran University of Medical Sciences, Tehran, Iran; 10 Faculty of Business and Law, Taylor’s University, Subang Jaya, Malaysia; 11 The Centre for Positive Psychology, Melbourne Graduate School of Education, University of Melbourne, Parkville, Australia; 12 Li Ka Shing Knowledge Institute, St. Michael’s Hospital, Unity Health Toronto, Toronto, Canada

**Keywords:** Attachment, Hope, Life satisfaction, Religiosity, Older adults, ageing

## Abstract

**Background:**

Attachment and support from family and friends are core to the experiences of ageing for older adults. The purpose of this study is to examine the relationships between attachment styles and hope, religiosity, and life satisfaction and provide new knowledge that may assist future planning for a rapidly ageing global population.

**Methods:**

In this cross-sectional study, 504 Iranian older adult participants from Qazvin province were recruited between December 2015 and April 2016. They completed a questionnaire that included the Revised Adult Attachment Scale, the Life Satisfaction Index-Z, and the Herth Hope Index.

**Results:**

Participants in the study had a mean age of 66.20 years (SD: 5.76) and most of them were women (57.5%). A mediation model testing the direct relationships between attachment, hope, religiosity, and life satisfaction showed a positive relationship between close attachment and religiosity (*β* = .226, *p* < .001) and a negative relationship between anxiety attachment and religiosity (*β* =  − .229, *p* < .001). Religiosity was positively related to hope (*β* = .384, *p* < .01) and hope was related to life satisfaction (*β* = .448, *p* < .001). Religiosity and hope mediated the relationship between close attachment (*β* = .119, *p* < .001) and anxiety attachment (*β* =  − .056, *p* < .01) with life satisfaction. More specifically, while religiosity and hope fully mediated the relationship between close attachment and life satisfaction, they partially mediated the attachment anxiety-life satisfaction link.

**Conclusions:**

Findings of the study provide insight into only a narrow perspective of life satisfaction and attachment given the many and varied variables that influence these constructs. Future research is needed whereby other related variables are introduced into the model to be examined further.

## Introduction

For older individuals, aged 65 years and over, attachment to and support from family and friends are important factors that may influence their quality of life (QOL) [1]. However, attachment theories also state that each individual’s attachment style can affect their perception of their QOL [2]. The aim of the current study was to investigate the relationship between attachment styles with hope, religiosity, and life satisfaction among Iranian elders. These three variables were chosen for this study as all three have significant impact on the perception of QOL in Iran, specifically, and in other cultures, generally.

In Iran, the population of elderly people has been increasing annually and changes in the family (roles and structure) have led people to experience psychological distress in this age group. Considering the marked increase in the elderly population in Iran and the impact of attachment styles in this age group, assessment of the factors associated with attachment and life satisfaction among the older adults is important. In a study of Iranian elders, Yazdani-Charati et al. [3] concluded that happiness, self-esteem, and self-care behaviors predicted a strong attachment to family and friends. Moreover, attachment styles affected their attitude and feelings towards themselves, the changes related to ageing, and their socio-cultural relationships [4]. Adults who lack perceived attachment pattern are more likely to feel lonely and experience depression compared to others [3,5].

Hope is defined as the ability to see a desirable outcome as a genuine possibility [6]. Adults in older age groups tend to be less hopeful and satisfied with their lives compared to younger individuals [7]. Findings of McGill, Paul [8] indicated that when physical health status diminisheded in  older age, feelings of hope also diminish. A person’s ability to be hopefulness ultimately influences life satisfaction [9]. Snyder suggests that attachments formed in early life can predict levels of hope later in life. Healthy relationships contribute to an individuals’ capacity to focus on goal-directed thoughts and general hopefulness [10]. When a person experiences grief for example, social support from peers has been found to be beneficial [11]. Hope has been identified as an essential element in effective psychological interventions. Facilitating hope in a therapeutic way involves constructing goals in the context of a collaborative therapeutic relationship [12]. Older adults  have a greater risk of psychological problems due to various agents (e.g. physical changes, loss of social support) compared to other age groups [13].

Although debate exists surrounding the differences between religiosity and spirituality, scholars unanimously agree that both constructs have some impact on individual’s attitudes, behaviors, and well-being [14]. In addition, religious practices (i.e., social or individual) encourage spiritual growth in adults [14] while beliefs about religion can inform the nature of attachments one may have with others [15]. Religious and spiritual beliefs have been found to have important implications for an individual’s ability to cope with adversity, which in turn has positive implications for physical and mental health [8]. Previous studies have shown that religion is positively related to happiness and reduced stress [16,17]. This is more evident in non-western countries as religion can play a more significant role in the cultural and social aspects of life for people living in those countries [18]. In the case of Iranian elders, studies have determined that religious beliefs can reduce death anxiety [19] and can be an essential aspect of an older adult's sense of power [20]. Religion has also been found to facilitate feelings of  hope towards the future [21,22]. It seems that by boosting hope, life satisfaction is improved [23,24].

Life satisfaction is defined as the cognitive appraisal of one’s satisfaction with life as a whole [25]. Kelley-Gillespie, Farley [26] found that older adults and their families had a higher QOL and life satisfaction once they moved from a nursing home to an assisted living facility. This finding provides insights into other significant factors that may influence older adult’s satisfaction in life (for example, independence, increased social support) [27]. A decline in social connections and physical functioning are the most widely reported negative aspects of ageing among the older age population [28]. Research indicates that individuals tend to interpret their life situation in terms of their own ageing process. This means that ageing can be considered a unique experience to each individual, often related to their attachment style.

 The attachment styles explores in the current study include: close (or secure), dependant/avoidant, and anxious. A close attachment style, which represents a secure attachment, is defined as the degree of comfort and intimacy that individuals perceive from an established relationship [2]. It is possible that the care and nurturing that people with a secure attachment style may have received  facilites them to have a more positive view toward themselves and others. As a result, they may have a greater sense of self-worth and trust in others compared to those without a secure attachment style. They may also be more equipped to establish a positive attitude towards social relationships [29].

The second attachment style investigated in the current study is dependant/avoidant. A dependant style is defined as the extent to which people feel comfortable depending on others and having partners depend on them [31]. While, people with an avoidant attachment style often avoid intimacy and closeness with others. Social communication, particularly in respect to discussing empotions, can be challenging for people with avoidant-type attachment [29,30]. When facing stressful situations, people with avoidant attachment can dismiss problems and and suppress their emotions which can minimizes the support they receive from, and provide to, others [29].

The third attachment style observed in this study is anxious. People with an anxious attachment style may show great respect for others, while not having a positive view of themselves [29]. This kind of attachment style in older adults has been speculated to have genetic roots and also be driven by  interactions with people who use both punitive and nurturing methods of care. These people could be considered to be between two states of being loved and rejected, and they can be skeptical about their sense of self-worth. As a result, older adults with anxious attachment styles can be overly reliant on the acceptance and approval of others. They can also be overly concerned about what other people think about them and, as a consequence, be reluctant to seek help [29].

 Older adults with secure attachment have been found to have higher social status, while those with avoidant attachment styles report higher scores in shyness, emotional instability, intensity of emotion, anger, and humiliation [31]. Loss of attachment with loved ones or loss of significant attachments can lead to prolonged distress and disability [32]. These issues, which may result from mental health problems, are associated with reduced cognitive capacity related to the ability to solve problems  and process information [33]. In a review of published research about attachment styles in Iran, Yazdani-Charati et al. [3] showed that insecure attachment style was related to an increased number of problems among older adults. Older individuals with secure attachment styles living at home had better health status compared to those living in nursing homes who experienced more anxiety, chronic disease, and sleep problems. Rezaei, Abdolahi, Akbari Balootbangan, Kheirkhahan [4] showed the significant differences among older adults living in nursing homes and sanatoriums  with avoidant, secure and anxiousety attachment styles.  Avoidant and anxious attachments of older adults in sanatoriums were higher than older adults living at home who reported higher secure attatchement scores.  

Various studies have examined the relationship between secure attachment style and hope. Simmons et al. [34] found that secure attachment style has a positive and significant relationship with trust and hope among adults. It was also found that those who have a secure attachment style through internal regulatory mechanisms are more flexible and constructive in their inter-personal relationships, and are able to build trust-based relationships and find ways to reach valuable goals in their lives. Based on the definition of hope, one can expect people with this style to be more interested in attachment with others, more hopeful and have more positive attitudes towards their goals. Previous researches on nurses [34], and students [12] found a significant relationship between attachment and hope. Other studies suggest that people with secure attachment are less likely to develop symptoms of depression [35,36], and therefore, will be more hopeful.

In relation to older adults, studies show that secure attachment styles can be a predictor of happiness [37], and happiness and hope in old age are related constructs [38]. Studies on the relationship between hope and attachment in healthy older adults are limited. In a sample, that compared older adults with mild cognitive impairment (MCI), mild dementia and moderate dementia with healthy participants, the healthy older adults had higher spiritual well-being, social support, self-esteem, life satisfaction, positive affect, optimism and hope scores, and lower negative affect scores compared to those in the other group  [39]. A randomized control study by Wu and Koo [40] found that a 6-week spiritual reminiscence intervention positively affected hope, life satisfaction, and spiritual well-being of older adult patients with mild or moderate dementia. Older adults who experience more life dissatisfaction, hopelessness, and helplessness may be at increased risk of depression and suicide [41].

Kirkpatrick [42] found that people with secure attachment had significantly higher scores than those with avoidant styles for religiousity. In addition, those with an avoidant attachment style were more likely to describe themselves as impious. According to Kirkpatrick [42], there are many factors influencing religious phenomena that cannot be justified by attachment styles only.

Life satisfaction is a cognitive evaluation of one's own life as a whole [43]. Several studies confirm that a safe and secure attachment style increases satisfaction in life [44,45]. Attachment styles affect a range of behaviors in older age groups, such as adaptation to chronic diseases, processes related to grief and loss, and overall life satisfaction [46]. Thompson and Ciechanowski [47] found that with increasing the advancement of age, secure attachment becomes one of the existential characteristics of an older adult. People with a secure attachment style have a more positive life. They also experience more empathy and support than others which leads to health promotion and satisfaction in all aspects of the individual's life [48].

To date, the current study is the first to analyze the different variables associated with life satisfaction and the mediating effects of religiosity and hope among older adults. The purpose of this study is to investigate the relationship of attachment with hope, religiosity, and life satisfaction. In view of this article’s goals, the hypotheses are that there will be a positive relationship between the secure attachment style and each variable and a negative relationship between dependant and anxietious attachment styles and each variable. The study will also examine the possible relationship between the variables of hope and religiosity with life satisfaction to see if there is a mediating affect.

## Materials and methods

### Data source

According to the results of power analysis using G*Power version 3.1.9.6, considering alpha of 0.05, power of 0.95, and effect size f^2^ of 0.04 with five predictors, the minimum required sample size in this cross-sectional study was 501 samples. Thus, the sample size of 504 participants which were recruited from Qazvin province between December 2015 and April 2016 was sufficient. A non-probability sampling method was used for data gathering. The inclusion criteria for participation in the study included participants who were: (1) willing to participate in the research; (2) aged 60 years and over; (3) aware of time and place; (4) able to communicate; and (5) able to respond to questionnaires. The participants included in this study were from health centers, clinics and public places (i.e. parks, culture centers). Participants who reported experiencing extreme stress in the past month and those with psychological problems were excluded. All participants were given fifteen minutes to finish the questionnaires and support was available to those participants who experienced difficulties with literacy.  

### Measures

The questionnaire consists of four parts: (1) Demographic information; (2) Adult Attachment Scale Revised (RAAS); (3) Life Satisfaction Index Z (LISZ); and (4) Herth Hope Index (HHI).

**Adult Attachment Scale Revised (RAAS).** The RAAS contains three subscales, each composed of six items. The three subscales are *close* (items 1, 6, 8, 12, 13, 17), *depend* (items 2, 5, 7, 14, 16, 18), and *anxiety* (items 3, 4, 9, 10, 11, 15). Items 2, 7, 8, 13, 16, 17, and 18 have reverse scores. The close scale measures the extent to which a person is comfortable with closeness and intimacy. The depend scale measures the extent to which a person feels they can depend on others to be available when needed. The anxiety attachment subscale measures the extent to which a person is worried about being rejected or unloved [49–51]. The reliability (α = 0.72 to 0.84) and validity of a translated version of this scale has been demonstrated in the Persian language [52]. As this version has not been used in Iran, the authors used content and face validity to validate it further. The Persian version of RAAS was evaluated by 10 experts (4 practitioner nurses, 2 nursing doctorates, 2 psychiatrists, and 2 clinical psychologists). The experts were asked to assess and comment on the wording, item allocation, and scaling of the items. They provided feedback regarding discrepancies found in certain items between the English and the Persian versions. Based on their comments, a final translation was created. Reliability was also assessed using Cronbach’s alpha which was reported to be acceptable (α = 0.71 to 0.79).

**Life Satisfaction Index Z (LISZ).** The LISZ is a shortened version of the Life Satisfaction Index A [53] with 13 items. The total score ranges from 0 to 26, with a higher score indicating higher overall life satisfaction [54]. The validity and reliability of this index has been demonstrated among older adults in Iran [55]. Cronbach’s alpha was 0.78 in the present study.

**Herth Hope Index (HHI).** The HHI is a 12-item abbreviated version of the Herth Hope Scale (HHS) measuring multidimensional aspects of hope based upon Dufault and Martocchio’s (1985) conceptual framework. It uses a 4-point Likert scale to assess the participant’s level of hope. The total HHI score ranges from 12 to 48 with higher scores corresponding to higher levels of hope [56]. The HHI has been used in studies worldwide with individuals experiencing varied health conditions in both hospital and community settings. To ensure the correct translation of the questionnaires, content validity was assessed. The reliability (α = 0.856 and 0.878) and validity of this scale has been demonstrated in the Persian language [57,58].

**Religiosity.** The measure of religiosity was adapted and simplified from empirically validated survey scales developed by nursing researchers to prevent potential burden to respondents due to their age [59]. Participants were asked to rate each item in the questionnaire using a 10-point Likert-type scale. For example, participants were required to evaluate the strength of their religious belief from 1 to 10 (1 = the weakest, 10 = the strongest).

### Statistical analysis

SPSS version 20 was used to summarize socio-demographic characteristics of the participants. Categorical variables and continuous variables were summarized using frequencies and percentages as well as mean and standard deviations (SD), respectively. The missing data were replaced using the mean imputation method [60]. The relationship between the research variables (i.e. the three dimensions of adult attachment, religiosity, hope, and life satisfaction) were examined by conducting Pearson correlation analysis.

The current study followed the two-step approach suggested by Hayes (2013) to investigate the research serial multiple mediation model [61]. First, the direct relationships between the three dimensions of adult attachment (close, depended, and anxiety) and life satisfaction without including religiosity and hope were tested (total effects model). Second, the mediators (i.e. religiosity and hope) were added to the model to develop a serial multiple mediation model (mediation effects model). In this model, close, dependent, and anxiety attachment as antecedent variables were modeled influencing life satisfaction directly as well as indirectly through the two mediators including religiosity and hope. The model was assessed using AMOS version 24 software. All path coefficients were estimated using maximum likelihood method based on the multivariate normality of observable variables and their significance was assessed using a bootstrapping with 2000 replications [62]. Next, the standard error of the indirect relationships was estimated by conducting the bootstrapping approach [60]. Bootstrapping is more accurate and has higher statistical power than Baron and Kenny (1986) and Sobel (1982) approaches [63,64]. The coefficient of determination (R^2^) was computed to assess how well the model explains the outcome.

## Results

Table 1 presents the demographic characteristics of participants in the study. The mean age for men in the study was 69.50 (SD: 0.50) and 66.24 (SD: 0.37) for women. Most of the participants were women (57.5%). Among the participants, 386 (76.6%) were married and 300 (59.5%) had guidance (or middle school) education level. Other descriptive statistics are presented in Table 1.

**Table 1:** Demographic profiles of respondents

Variables	N (%)	Variables	N (%)
*Sex*		*Present socio-economic status*	
Male	214 (42.5%)	Poor	85 (16.9%)
Female	290 (57.5%)	Intermediate	334 (66.3%)
*Age*	66.20 (5.76)	Good	85 (16.8%)
*Marital status*		*Main ıncome resources*	
Single	27 (5.4%)	Personal	170 (33.7%)
Married	386 (76.5%)	Family	77 (15.3%)
Widow/divorced	91 (18.1%)	Pension	257 (51%)
*Educational status*		*Relative visiting*	
Illiterate	119 (23.6%)	Sometimes	253 (50.2%)
Guidance	300 (59.5%)	Often	157 (31.2%)
Diploma	56 (11.1%)	Very much	94 (18.6%)
Collegiate	29 (5.8%)	*Emotional support*	
*Present living place*		Family	475 (94.2%)
Personal	463 (91.9%)	Friends and colleagues	29 (5.8%)
Children	41 (8.1%)		

Table 2 shows the results of conducting Pearson correlation analysis and descriptive statistics. Close had a significant positive relationship with religiosity (r = 0.127, *p* < 0.01), hope (r = 0.234, *p* < 0.01), and life satisfaction (r = 0.094, *p* < 0.05). Anxiety attachment was negatively correlated with religiosity (r =  − 0.160, *p* < 0.01) and life satisfaction (r =  − 0.138, *p* < 0.01). However, this study could not find any significant relationship between anxiety attachment and hope (r =  − 0.051, *p* = 0.255). Moreover, no significant association was found between depend with religiosity (r =  − 0.045, *p* = 0.318), hope (r =  − 0.022, *p* = 0.622), and life satisfaction (r =  − 0.073, *p* = 0.103).

**Table 2:** Correlation analysis results

	Mean	SD	[2]	[3]	[4]	[5]	[6]
1. Close	3.117	0.391	.356**	.387**	.127**	.234**	.094*
2. Depend	3.003	0.433		.438**	− .045^*ns*^	.022^*ns*^	− .073^*ns*^
3. Anxiety	2.777	0.693			− .160**	− .051^*ns*^	− .138**
4. Religiosity	23.512	3.403				.420**	.149**
5. Hope	35.663	5.284					.438**
6. Life satisfaction	40.238	6.380					

^*ns*^*p* ≥ .05, *p < .05, **p < .01, two-tailed tests

**Fig. 1:**
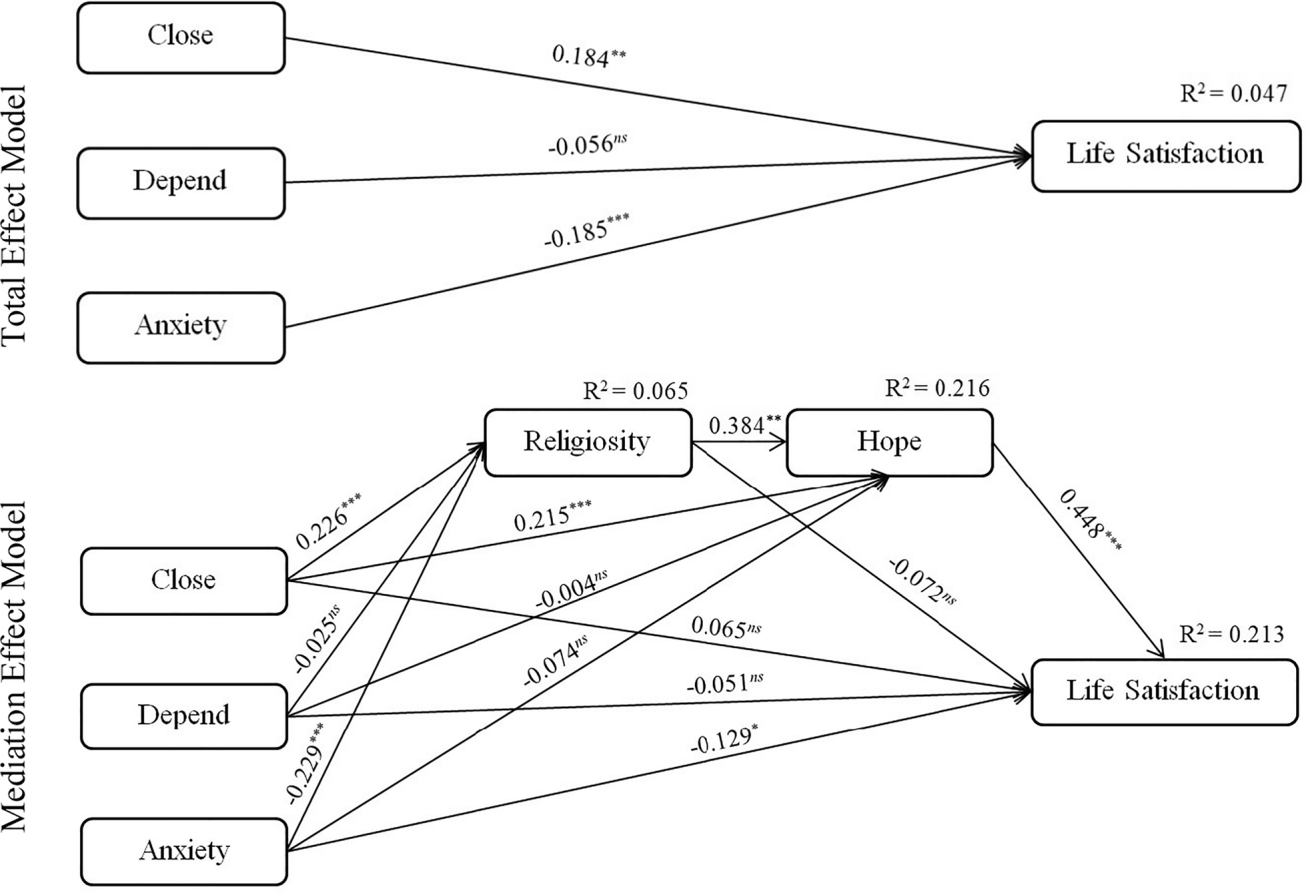
Total effect and mediation effect models

Following Hayes (2013), this study assessed the developed serial multiple mediation model and tested the research hypotheses by conducting bootstrapping with 2000 replications [61]. Standardized path coefficients of total and mediation effects models and their 95% confidence intervals are shown in Fig. 1 and reported in Table 3 as well. The results of assessing the total effect model showed a significant relationship between close (*β* = 0.184, *p* < 0.01) and anxiety attachment (*β* =  − 0.185, *p* < 0.001) with life satisfaction. However, the results could not support the relationship between depend and life satisfaction (*β* =  − 0.056, *p* = 0.320). The results allowed this study to test the serial multiple mediation model for the relationship between close and anxiety attachment with life satisfaction. The results of testing the direct relationships of the mediation model effect showed a positive relationship between close and religiosity (*β* = 0.226, *p* < 0.001). Also, a negative relationship between anxiety attachment and religiosity (*β* =  − 0.229, *p* < 0.001) was found. Religiosity was positively related to hope (*β* = 0.384, *p* < 0.01) and hope was related to life satisfaction (*β* = 0.448, *p* < 0.001). However, this study failed to support the association between depend and religiosity (*β* =  − 0.025, *p* = 0.697). Religiosity and hope mediated the relationship between close (*β* = 0.119, *p* < 0.001) and anxiety attachment (*β* =  − 0.056, *p* < 0.01) with life satisfaction. More specifically, while religiosity and hope fully mediated the relationship between close and life satisfaction, they partially mediated the anxiety-life satisfaction link. This was because the direct association between close and life satisfaction in the mediation effects model was not significant (*β* = 0.065, *p* = 0.189) whereas the link between anxiety and life satisfaction was significant (*β* =  − 0.129, *p* < 0.01). Finally, this study could not support the indirect relationship between depend and life satisfaction through religiosity and hope (*β* =  − 0.004, *p* = 0.865). The model explained 6.5% of the variance of religiosity, 21.6% of the variance of hope, and 21.3% of the variance of life satisfaction.

**Table 3:** Direct, indirect, and total effects

Path	Standardized path coefficients	95% confidence level	
		Lower bound	Upper bound
*Total effects*			
Close → Life satisfaction	.184**	− .300	− .058
Depend → Life satisfaction	− .056^*ns*^	− .161	.055
Anxiety → Life satisfaction	− .185***	.085	.287
*Direct effects*			
Close → Religiosity	.226***	.129	.330
Depend → Religiosity	− .025^*ns*^	− .129	.086
Anxiety → Religiosity	− .229***	− .322	− .131
Close → Hope	.215***	.112	.300
Depend → Hope	− .004^*ns*^	− .094	.085
Anxiety → Hope	− .074^*ns*^	− .162	.018
Close → Life satisfaction	.065^*ns*^	− .032	.164
Depend → Life satisfaction	− .051^*ns*^	− .151	.050
Anxiety → Life satisfaction	− .129*	− .235	− .011
Religiosity → Hope	.384**	.284	.461
Religiosity → Life satisfaction	− .072^*ns*^	− .153	.029
Hope → Life satisfaction	.448***	.356	.522
*Indirect effects*			
Close → Life satisfaction	.119***	.073	.170
Depend → Life satisfaction	− .004^*ns*^	− .047	.039
Anxiety → Life satisfaction	− .056**	− .101	− .014

^*ns*^*p* ≥ .05, *p < .05, **p < .01, *** *p* < .001, two-tailed tests

## Discussion

The aim of the current study was to investigate the relationship between attachment with hope, religiosity, and life satisfaction among older adults living in Iran. One of the main results of the present study was the direct relationship of close attachment with religiosity, hope, and life satisfaction. The current research presents the first data regarding the role of hope, life satisfaction, and religiosity as the mediators of older adult's close and anxious attachment styles.

The current study aligns with previous research in finding a positive correlation between secure (i.e., close) attachment styles and hope and a negative correlation between anxious attachment styles and hope. While happiness is identified as a predictor of hope, studies have revealed that it is the main unmet need in Iranian older adults [65,66]. According to the *World Happiness Report* in 2020, Iran ranked 118 out of 153 based on the countries included in the study, reporting an average score of 4.67, below the global average of 5.48 [67]. Further, studies in Iran have been determined a high prevelance of mental health problems in older adults that have resulted from care neglect and rejection [68]. This might be one reason why hope has been identified as an important factor predicting positive perceptions of ageing in older adults in Iran [9], and their ability to cope with challenges and threats to their well-being [69].

The present study identified a positive correlation between a close and secure attachment style and life satisfaction, communication with family members, and receiving support from them. This finding also aligns with previous research. About 70% of older adults in the present study have been in recent contact with their relatives. Also, about 95% of them had family support. The current finding could be a  reflection of  cultural norms and expectations of the Iranian population. As is the case for traditional families, Iranian individuals’ life can be centred around  family relationships. Imanzadeh & Hamrahzade in their phenomenological study found that visiting relatives was an important way of improving quality of life in older adults [70]. Studies show that frequent visits, especially with family members and receiving social support, facilitates happiness, promotes mental health, and ultimately leads to increased satisfaction with life [71,72].

The results of the study showed that anxious attachment style has a reverse and significant relationship with attachment to religion and life satisfaction as expected from previous research. Also, results of previous studies have highlighted the positive relationship between religion and life satisfaction. Krause, Hayward [73] found that the expression of feelings and emotions during worship for those who have religious beliefs predicts the relationship between religion and life satisfaction. Some research has showed that negative attitudes toward religion is negatively associated with life satisfaction in China and Vietnam [74–76].

One of the most important results of the present study is the effect of introducing the two variables of religion and hope in the model as a mediator. Comparing the value between the two models shows that adding religion and hope in the model leads to a better understanding of the relationship between attachment styles and life satisfaction.

The results of this study demonstrate that there is an inverse association between the dependant/avoidant attachment style and hope, religion, and life satisfaction variables, but this correlation is not statistically significant.

### Limitations

This study had some limitations. Data collection tools may have offered some challenges for the older aged participants of the study. It is unclear whether the participants answered honestly or whether they had a good overall understanding of the items in the questionnaires (as nearly 25% presented with difficulties with literacy). Another limitation of the current study is that the participant group was drawn from the residents of the same city, thus, the present results cannot be generalized to a larger older adult population in Iran. Despite these limitations, this study provides useful insights into the attachment styles of older adults in Iran, especially considering that research in this area is limited.

## Future directions

Quantitative analysis focused on attachment, hope, life satisfaction, and religiosity is recommended for future research. Moreover, other factors (i.e. education, past occupation, housing, inequality) that may have affected the main variables of the current research should be assessed in future studies. Case studies may also shed light on the perceptions of attachment among older adults. An important finding from the study was the role of hope and religiosity for life satisfaction in the participants, which holds merit for clinical practice of nurses, psychologists and other health care professionals. Future research may then focus on how hope and religiosity can be used as an intervention to increase life satisfaction in older people and investigate how this may affect their overall physical and mental well-being.

### Implications

The psychological variables identified in this study together with the overall findings have implications for health practitioners in identifying interventions to improve the mental health status and QOL of the older age population. Future research is needed whereby other related variables (e.g., sense of belonging, happiness, meaningful life) are introduced into the model of the present study.  

## Conclusions

Based on the results of this study, religiosity was positively related to hope and hope was positively related to life satisfaction. Religiosity and hope mediated the relationship between close and anxious attachment styles with life satisfaction. Since many variables affect both life satisfaction and attachment styles, it is suggested that future research is needed. 

## Data Availability

Available data are presented in the manuscript.

## Abbreviations

QOL: Quality of Life
MCI: Mild Cognitive Impairment
RAAS: Adult Attachment Scale Revised
LISZ: Life Satisfaction Index Z
HHI: Herth Hope Index
SD: Standard Deviation
